# Ecological reshaping of pediatric respiratory pathogen profiles after COVID-19-related non-pharmaceutical interventions were lifted: a 5-year interrupted time-series study

**DOI:** 10.3389/fped.2026.1889078

**Published:** 2026-09-22

**Authors:** Lijun Du, Bing Zhang, Xin Li, Li Li, Miyuan Yang, Zhihua Zuo, Honglian Yuan, Jie Wang, Yan Xing

**Affiliations:** 1 Department of Clinical Laboratory, Beijing Anzhen Nanchong Hospital of Capital Medical University and Nanchong Central Hospital, Nanchong, Sichuan, China; 2 Department of Clinical Laboratory, The Second Clinical Medical College of North Sichuan Medical University, Nanchong, Sichuan, China

**Keywords:** co-detection, COVID-19, interrupted time-series analysis, laboratory surveillance, meteorological factors, non-pharmaceutical interventions, pediatric respiratory pathogens, school term

## Abstract

**Background:**

COVID-19-related non-pharmaceutical interventions (NPIs) disrupted the circulation of many respiratory pathogens. After these measures were lifted, pediatric respiratory pathogen activity may have changed in pathogen-specific ways. However, laboratory-based evidence using continuous multi-year data, interrupted time-series analysis, and contextual factors such as meteorology, air quality, and school calendars remains limited.

**Methods:**

We conducted a single-center retrospective laboratory study of pediatric and neonatal inpatients aged 0–14 years with clinically diagnosed lower respiratory tract infection from January 2021 to December 2025. After 7-day deduplication within the same child, 20,250 throat-swab testing records were included. The NPI period was January 2021–December 2022, and the post-NPI period was January 2023–December 2025. Thirteen pathogen categories were analyzed using period comparisons, interrupted time-series models, and multivariable logistic regression incorporating meteorological factors, air quality index, and school-term status.

**Results:**

Overall positivity was higher in the post-NPI period than in the NPI period (75.4% vs. 58.8%), and co-detection was also higher in the post-NPI period (15.2% vs. 6.0%). Pathogen-specific interrupted time-series analysis showed heterogeneous patterns: human adenovirus showed a robust immediate level decrease (−13.00 percentage points; 95% CI, −20.13 to −5.87; FDR-adjusted *P* = 0.005), whereas human rhinovirus showed a significant positive trend change after the January 2023 interruption (+1.07 percentage points per month; 95% CI, 0.63–1.50; FDR-adjusted *P* < 0.001), with a positive post-NPI slope (+0.64 percentage points per month). Higher temperature was associated with lower positivity of human respiratory syncytial virus, influenza A H1N1 (2009) virus, and influenza B virus, but higher positivity of human parainfluenza virus and human bocavirus. School-term status was associated with higher human rhinovirus positivity (aOR, 1.84; 95% CI, 1.34–2.52; FDR-adjusted *P* = 0.002).

**Conclusion:**

The NPI and post-NPI periods showed distinct and heterogeneous temporal patterns in pediatric respiratory pathogen detection. These findings support integrated hospital-based laboratory surveillance centered on pathogen-specific temporal trends and interpreted alongside demographic, multi-pathogen, environmental, school-calendar, and longer-term temporal context.

## 1 Introduction

Acute respiratory infections remain a major cause of illness among children worldwide and are associated with substantial healthcare use, hospitalization, and public health burden (1). Among specific pediatric pathogens, respiratory syncytial virus, seasonal influenza virus, human metapneumovirus, and human parainfluenza virus contribute substantially to acute lower respiratory infection burden in young children (2–5). Laboratory-based surveillance in China has also shown that pediatric and community respiratory specimens can involve diverse viral and atypical pathogens rather than a single dominant target (6). These patterns vary by age, season, geography, and social contact structure, highlighting the importance of continuous laboratory-based surveillance for understanding temporal changes in pediatric respiratory pathogen activity.

During the COVID-19 pandemic, non-pharmaceutical interventions (NPIs), such as mask wearing, school closures, reduced mobility, physical distancing, and enhanced hygiene measures, substantially altered community respiratory virus activity beyond SARS-CoV-2 (7). Observational and pediatric hospital-based studies in China showed broad impacts of NPIs on acute respiratory infections and common childhood respiratory viruses (8, 9). Respiratory syncytial virus-specific studies also reported altered epidemiological and clinical characteristics among hospitalized children during the pandemic period (10). These changes were not uniform across pathogens or regions, suggesting that pathogen biology, population susceptibility, testing behavior, and contact patterns may have interacted to shape observed laboratory detection trends.

After the relaxation or lifting of COVID-19-related NPIs, many settings reported renewed activity of pediatric respiratory pathogens, but post-NPI changes have not consistently followed a single synchronized rebound pattern. Studies have described off-season or asynchronous resurgence of influenza viruses, respiratory syncytial virus, human metapneumovirus, and other common respiratory viruses after periods of reduced activity (11–13). Post-NPI resurgence or shifting epidemic features of Mycoplasma pneumoniae have also been reported in China and related pediatric studies (14, 15). The concept of immune debt has further been proposed to help explain heterogeneous pediatric respiratory infection activity after the pandemic (16). These observations suggest that the post-NPI period may be better understood as a phase of pathogen-specific reshaping of respiratory pathogen detection patterns rather than a simple return to pre-pandemic seasonality.

In parallel with changes in population contact patterns, environmental and calendar-related factors may provide important context for respiratory pathogen activity. Temperature and meteorological conditions have been associated with viral seasonality and pathogen-specific acute respiratory infection patterns (17, 18). Air pollution has also been linked to respiratory infection susceptibility and pediatric respiratory health (19). School sessions have been associated with seasonal outbreaks of medically attended respiratory infections (20). Nevertheless, few studies have jointly evaluated meteorological conditions, air quality, and school-term status across multiple pediatric respiratory pathogen categories in the post-NPI period.

Although evidence on post-NPI respiratory pathogen activity is increasing, the available literature remains constrained by several methodological limitations. Many studies have relied on short observation windows, selected-pathogen analyses, or simple before-and-after comparisons, which are insufficient to separate an abrupt post-policy level change from a sustained change in temporal slope. Continuous multi-year laboratory datasets that span both the NPI and post-NPI periods and simultaneously evaluate multiple pediatric respiratory pathogens remain scarce. Moreover, environmental conditions, air quality, and school-calendar context are rarely assessed within the same pathogen-specific analytical framework. These limitations restrict the ability of surveillance studies to determine whether post-NPI changes represent a uniform rebound or a more complex reshaping of pathogen-specific detection patterns.

To address these gaps, we analyzed 20,250 pediatric throat-swab testing records collected over five consecutive years, from January 2021 to December 2025. We compared pathogen detection risks between the NPI and post-NPI periods, evaluated overall long-term and segmented temporal changes, applied pathogen-specific interrupted time-series models, and assessed associations of meteorological factors, air quality, and school-term status with pathogen-specific positivity, including potential variation in these associations across study periods. By integrating a large continuous laboratory dataset with time-series and contextual analyses, this study aimed to characterize pathogen-specific temporal patterns across the NPI and post-NPI periods and their relevance to hospital-based laboratory surveillance.

## 2 Materials and methods

### 2.1 Study design, setting, and data source

This single-center, laboratory-based retrospective observational study was conducted in the Department of Clinical Laboratory at Beijing Anzhen Nanchong Hospital of Capital Medical University and Nanchong Central Hospital, located in Nanchong, Sichuan Province, China. The hospital is a tertiary general hospital and clinical teaching hospital that receives patients from Nanchong and surrounding areas. Pediatric respiratory pathogen nucleic acid testing records from January 1, 2021, to December 31, 2025, were extracted from the laboratory information system. Throughout the study period, the data-collecting hospital, clinical laboratory, and respiratory pathogen testing framework remained consistent. Annual pediatric and neonatal inpatient volumes from 2021 to 2025 were obtained from hospital administrative records and used to calculate the number of respiratory pathogen testing records per 100 inpatient admissions as a contextual measure of testing volume across study periods.

### 2.2 Study population

Eligible testing records were from pediatric or neonatal inpatients aged 0–14 years with a clinician-recorded diagnosis of lower respiratory tract infection on the laboratory test request. These patients underwent routine 13-target multiplex respiratory pathogen testing between January 1, 2021, and December 31, 2025. Testing was ordered by clinicians according to the child's clinical presentation and diagnostic needs. Throat swabs were collected for respiratory pathogen nucleic acid testing. Records were required to contain complete information on sex, age, application date, and pathogen detection results. Outpatient and emergency department records were not included. Throughout the study period, the source wards, age range, eligibility criteria, and specimen type remained consistent.

A total of 21,132 original identification records were initially available. After preliminary data cleaning, variable harmonization, and verification of key fields, 20,296 preliminary testing records were retained. To reduce the influence of repeated short-term testing from the same child, 7-day deduplication was performed using medical record numbers. For each child, testing records were ordered chronologically by application time; when multiple respiratory pathogen tests occurred within a 7-day window, only the first record was retained, whereas a test performed more than 7 days after the previously retained record was considered a new testing event. After deduplication, 46 short-term duplicate records were removed, leaving 20,250 testing records for the main analysis, including 6,490 records from the NPI period and 13,760 from the post-NPI period. The final dataset covered 60 consecutive months from January 2021 to December 2025. The overall study design, data-cleaning process, 7-day deduplication procedure, study-period classification, and analytical workflow are summarized in Figure 1.

**Figure 1:**
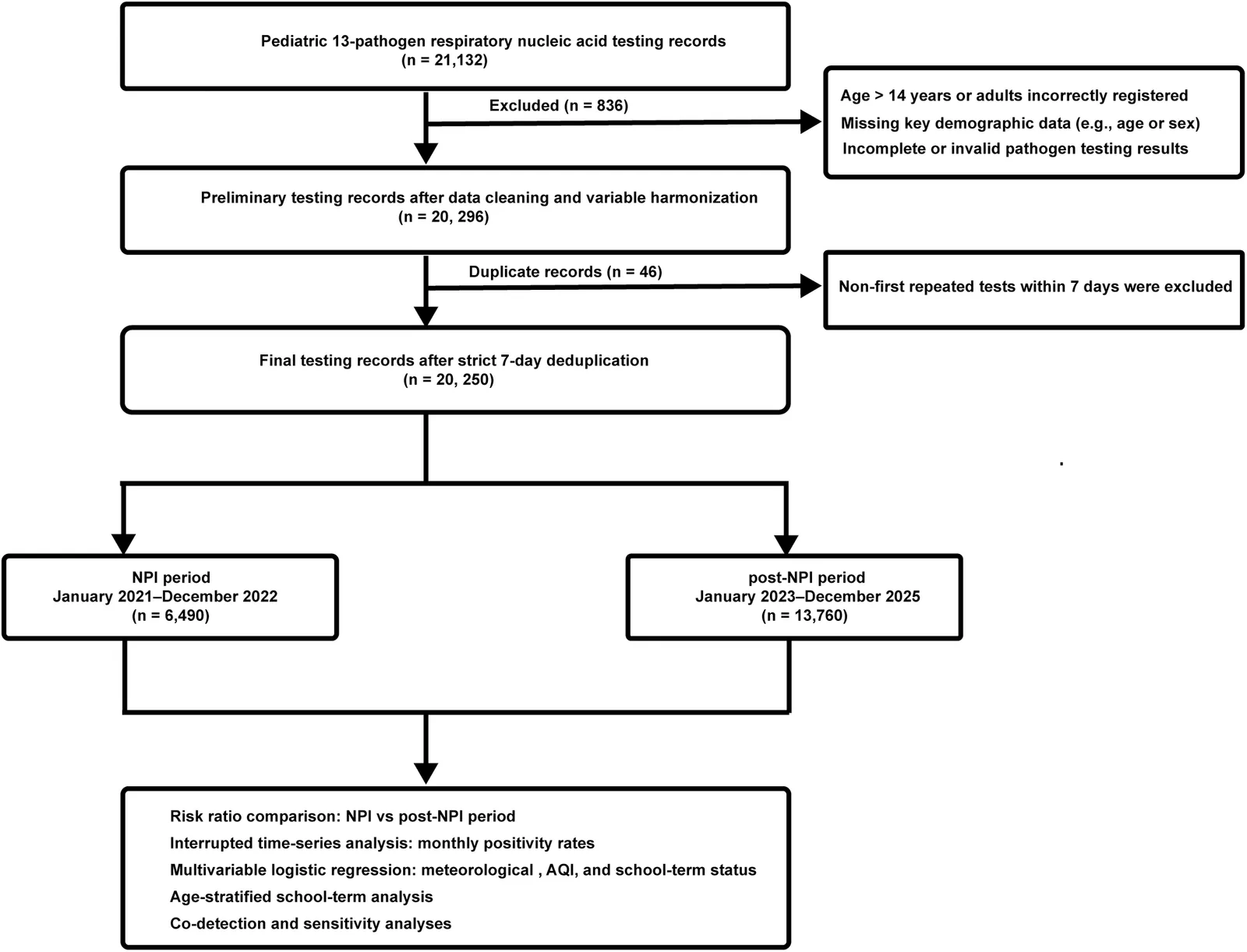
Study design, data cleaning, and analytical workflow. The figure shows the data source, exclusion process, 7-day deduplication, study-period classification, and analytical framework. The NPI period was defined as January 2021 to December 2022, and the post-NPI period was defined as January 2023 to December 2025. During 7-day deduplication, only the first testing record from the same child within a 7-day window was retained. NPI, non-pharmaceutical intervention.

Children were categorized into four age groups: <1, 1–<3, 3–<6, and 6–14 years. This age stratification was selected according to commonly used pediatric respiratory infection epidemiology and the social contact patterns of children in China, broadly corresponding to infancy, early childhood mainly under home care, kindergarten age, and school age.

### 2.3 Respiratory pathogen testing and pathogen definitions

Respiratory pathogen nucleic acid testing was performed using a multiplex detection kit for 13 respiratory pathogens based on fluorescence PCR followed by capillary electrophoresis (PN: 1060071; Health GeneTech Co., Ltd., Ningbo, China). Nucleic acid extraction was performed using a TANBead SLA-32 automated nucleic acid extraction system. PCR amplification was performed using a SLAN-96P real-time PCR system. Amplification products were detected using an Applied Biosystems 3500 Dx Genetic Analyzer for capillary electrophoresis-based detection and fragment analysis. Sample processing, nucleic acid extraction, PCR amplification, capillary electrophoresis, quality control, and result interpretation were performed according to the manufacturer's instructions and local standard operating procedures.

The main analysis included 13 respiratory pathogen categories: influenza A virus not further classified by this panel (InfA-NFC), influenza A H1N1 (2009) virus, seasonal influenza A (H3N2) virus, influenza B virus, human respiratory syncytial virus, human parainfluenza virus, human metapneumovirus, human rhinovirus, human adenovirus, seasonal human coronaviruses, human bocavirus, Mycoplasma pneumoniae, and Chlamydia spp. In this study, InfA-NFC referred to records positive for the manufacturer-reported generic influenza A target, listed as InfA in the manufacturer's instructions, but not classified as influenza A H1N1 (2009) virus or seasonal influenza A (H3N2) virus. The seasonal human coronaviruses category did not include SARS-CoV-2. Throughout the study period, SARS-CoV-2 testing, when clinically indicated, was performed through a separate dedicated nucleic acid testing pathway and was therefore not included in the fixed 13-target respiratory pathogen panel analyzed in this study. The Chlamydia category was not further differentiated into C. trachomatis or C. pneumoniae. Pathogen abbreviations, full pathogen names, manufacturer-reported detection targets, and limits of detection are provided in Supplementary Table S1.

A composite influenza A variable was defined as positivity for any influenza A-related analytic category, including InfA-NFC, influenza A H1N1 (2009) virus, or seasonal influenza A(H3N2) virus. This composite variable was used only for descriptive summaries and was not included as an additional category in the main risk ratio, multiple-comparison, interrupted time-series, or multivariable regression analyses to avoid double counting. Overall positivity was defined as positivity for at least one of the 13 main respiratory pathogen categories. Co-detection was defined as positivity for two or more of the 13 main pathogen categories in the same testing record. Positivity and co-detection rates were calculated using the corresponding number of testing records as the denominator. A 13-target-negative record was defined as a testing record in which none of the 13 main respiratory pathogen categories was detected. Overall positivity and 13-target-negative proportions describe laboratory detection among tested pediatric and neonatal inpatients and were not intended as estimates of community incidence or prevalence.

### 2.4 Study periods, seasons, and school-term status

The study period was divided according to the timing of COVID-19 policy adjustment in China and the monthly structure of the dataset. January 2021 to December 2022 was defined as the NPI period, and January 2023 to December 2025 was defined as the post-NPI period. In interrupted time-series analyses, January 2023 was treated as the first post-intervention month. Seasons were defined by calendar month: spring, March to May; summer, June to August; autumn, September to November; and winter, December to February. School-term status was defined at the calendar-day level according to the official local school calendar rather than by calendar month alone and assigned to each testing record based on the application date. Each testing record was classified as occurring during an in-school term or a vacation period. This variable was used as a population-level contextual indicator of periods with increased or decreased community contact networks related to kindergarten and school attendance.

### 2.5 Meteorological and air-quality data

Daily meteorological observations for Nanchong, Sichuan Province, China were obtained from RP5 (Reliable Prognosis), a publicly available weather data platform providing observational data from ground weather stations. Daily air quality index (AQI) records were obtained from the 2345 Weather historical weather database for Nanchong, a publicly accessible weather and air-quality information platform that provides city-level historical weather records with daily air-quality information (https://tianqi.2345.com/wea_history/57411.htm; accessed July 4, 2026). Before monthly aggregation, extracted AQI records were checked for completeness, date consistency, plausible value ranges, and temporal continuity. Daily temperature, relative humidity, and AQI records were aggregated into monthly means for each calendar month and linked to individual testing records by year-month. Temperature was modeled per 1 °C increase, relative humidity per 10% increase, and AQI per 10-unit increase. Meteorological and AQI variables were available for all 60 calendar months from January 2021 to December 2025 and were matched to the testing dataset by year-month.

### 2.6 Statistical analysis

All statistical analyses were based on the final analytic dataset of 20,250 testing records. Categorical variables were summarized as counts and percentages. Continuous variables were summarized as medians with interquartile ranges or means with standard deviations, as appropriate. All tests were two-sided, and *P* < 0.05 was considered statistically significant. For multiple comparisons across the 13 main pathogen categories, the Benjamini–Hochberg method was used to control the false discovery rate (FDR). All statistical analyses were performed using Python software. Data management and aggregation were conducted using pandas and NumPy. Statistical calculations and modeling were performed using SciPy and statsmodels, and figures were generated using matplotlib.

#### 2.6.1 Baseline characteristics and period comparisons

Baseline characteristics and pathogen detection profiles were compared between the NPI and post-NPI periods. Categorical variables were compared using the *χ*^2^ test or Fisher's exact test, as appropriate. Continuous variables were compared using the Mann–Whitney *U* test or *t* test, depending on distributional characteristics. The composite influenza A variable was presented only as a descriptive summary, with influenza A subtypes listed separately. Continuous age is presented as median (interquartile range) and was compared between the NPI and post-NPI periods using the Mann–Whitney *U* test.

#### 2.6.2 Risk ratio analysis

Detection risks for the 13 main respiratory pathogen categories were compared between the NPI and post-NPI periods. For each pathogen, positivity rates were calculated for both periods, and risk ratios (RRs) with 95% confidence intervals (CIs) were estimated. FDR-adjusted *P* values were used to assess statistical robustness under multiple testing. As an additional RSV-focused supplementary analysis, age was reclassified into clinically relevant categories for RSV burden and prevention strategies. RSV detection was first compared between children aged <2 years and those aged ≥2 years. Children were further categorized as 0–<6 months, 6–<12 months, 12–<24 months, and ≥24 months. For each category, RSV positivity was summarized for the NPI and post-NPI periods, and post-NPI vs. NPI risk ratios (RRs) with 95% confidence intervals (CIs), *P* values, and FDR-adjusted *P* values were calculated. This RSV-specific supplementary analysis was designed to provide clinical and public-health detail for the age windows most relevant to infant and toddler RSV prevention.

#### 2.6.3 Interrupted time-series analysis

To evaluate whether the period difference in overall positivity could reflect a general long-term trend, monthly overall positivity was first analyzed using a single time-trend model without an interruption term. A segmented interrupted time-series model was then fitted using January 2023 as the first post-interruption month, corresponding to the January 8, 2023 policy transition, to estimate the pre-interruption trend, immediate level change, and post-interruption trend change. A joint Wald test evaluated the combined evidence for the immediate level and trend changes, and a grouped binomial model based on monthly positive and negative counts was used as a sensitivity analysis.

Interrupted time-series (ITS) segmented regression models were used to assess immediate level changes and post-intervention trend changes in monthly pathogen positivity after the lifting of NPIs. ITS and segmented regression models are commonly used to evaluate level and slope changes after public health policy or intervention changes (21–24).

For each pathogen, the monthly positivity rate was used as the outcome. The core independent variables included time, intervention status, and time after intervention. Month fixed effects were included to account for seasonality. Models were weighted by the monthly number of testing records. Newey–West robust standard errors were used to account for potential autocorrelation (25). The primary model used Newey–West lag = 1, and lag = 3 was used as a sensitivity analysis.

The model was specified as: *Y_t_* = *β*_0_ + *β*_1_ × time + *β*_2_ × intervention + *β*_3_ × post-intervention time + month fixed effects + *ε_t_*, where *β*_1_ represents the pre-intervention monthly trend, *β*_2_ represents the immediate level change after the lifting of NPIs, and *β*_3_ represents the post-intervention trend change.

#### 2.6.4 Multivariable logistic regression

To evaluate factors associated with pathogen positivity, separate multivariable logistic regression models were fitted for each of the 13 main respiratory pathogen categories. The core independent variables were monthly mean temperature, monthly mean relative humidity, monthly mean AQI, and school-term status. Models were adjusted for age group, sex, and pandemic phase. Because meteorological exposures, seasonality, and time trends may be correlated, season was not included in the primary model to reduce potential collinearity; this approach was consistent with general considerations in environmental time-series modeling (26). Cluster-robust standard errors were estimated at the year-month level. Results were reported as adjusted odds ratios (aORs) with 95% CIs. To assess whether environmental and temporal associations differed between the NPI and post-NPI periods, separate interaction models were fitted by adding temperature × phase, relative humidity × phase, AQI × phase, and school term × phase terms to the pathogen-specific multivariable models. Across 13 pathogen categories and four contextual factors, 52 interaction tests were prespecified. Benjamini–Hochberg FDR correction was applied to all estimable interaction *P* values; for interactions remaining significant after FDR correction, phase-specific adjusted effect estimates were subsequently calculated.

To further assess age heterogeneity in the association between school-term status and pathogen positivity, age-stratified logistic regression models were fitted for three age strata: <3, 3–<6, and 6–14 years. Within each age stratum, school-term status was the main exposure, and models were adjusted for sex, pandemic phase, and monthly mean meteorological and air-quality variables, including temperature, relative humidity, and AQI. Cluster-robust standard errors were estimated at the year-month level, and within each age stratum, Benjamini–Hochberg FDR correction was applied across the 13 pathogen-specific school-term comparisons.

#### 2.6.5 Co-detection analysis

Co-detection was defined as positivity for at least two of the 13 main respiratory pathogen categories in the same testing record. Co-detection rates were compared between the NPI and post-NPI periods, and RRs with 95% CIs were calculated. Monthly co-detection rates were further evaluated using the same ITS framework applied to pathogen-specific monthly positivity. Co-detection analyses were presented as supplementary analyses.

#### 2.6.6 Sensitivity and robustness analyses

Several sensitivity analyses were conducted to assess the robustness of the main findings. First, to evaluate whether ITS findings were influenced by the policy transition period, ITS models were refitted after excluding December 2022 and January 2023, and again after excluding December 2022 to February 2023. These analyses assessed whether immediate level changes and post-intervention trend changes were robust to the exclusion of transition months (27–29). Second, because monthly positivity rates were derived from monthly positive and negative counts, binomial ITS models were fitted as an additional sensitivity analysis. These models used monthly positive and negative counts as outcomes and included time, intervention status, time after intervention, and month fixed effects, thereby assessing whether pathogen-specific findings were consistent under a count-based modeling framework (27, 29).

To evaluate collinearity in the multivariable models, correlation matrices and variance inflation factors were calculated for the core predictors, including school-term status and monthly mean meteorological and air-quality variables: temperature, relative humidity, and AQI. A season-adjusted sensitivity model was also fitted by adding season to the primary multivariable logistic regression model, and the direction and statistical significance of core associations were compared between the primary and season-adjusted models (26). Finally, supplementary co-detection analyses included RR analysis, weighted ITS analysis, and binomial ITS analysis for monthly co-detection rates.

To assess the influence of children contributing multiple testing records across the 5-year study period, a first-record-only sensitivity analysis was also performed. For each unique child identified by medical record number, only the earliest testing record in the entire study period was retained, irrespective of whether it occurred during the NPI or post-NPI period. Overall positivity, co-detection, and the detection risks of the 13 pathogen categories were then recalculated using the same period-comparison methods as in the main analysis, including Benjamini–Hochberg FDR correction for the 13 pathogen-specific comparisons.

## 3 Results

### 3.1 Study population and baseline characteristics

A total of 20,250 throat-swab testing records were included in the main analysis, covering 60 consecutive months from January 2021 to December 2025, including 6,490 records during the NPI period and 13,760 during the post-NPI period. Monthly testing volume and overall positivity trends are shown in Figure 2. Among the included records, 11,191 were from boys (55.3%) and 9,059 from girls (44.7%). The median age was 2.00 years overall and in both the NPI and post-NPI periods. The overall IQR was 0.75–4.00 years, while the IQRs were 0.67–4.00 years during the NPI period and 0.75–4.00 years during the post-NPI period. The largest age group was 3–<6 years (30.2%), followed by <1 year (29.4%), 1–<3 years (25.3%), and 6–14 years (15.1%). Overall, 16,871 records (83.3%) were collected during school terms and 3,379 (16.7%) during vacation periods. Baseline demographic and temporal characteristics differed between the NPI and post-NPI periods, including sex distribution, age group, season, and school-term status (Table 1).

**Figure 2:**
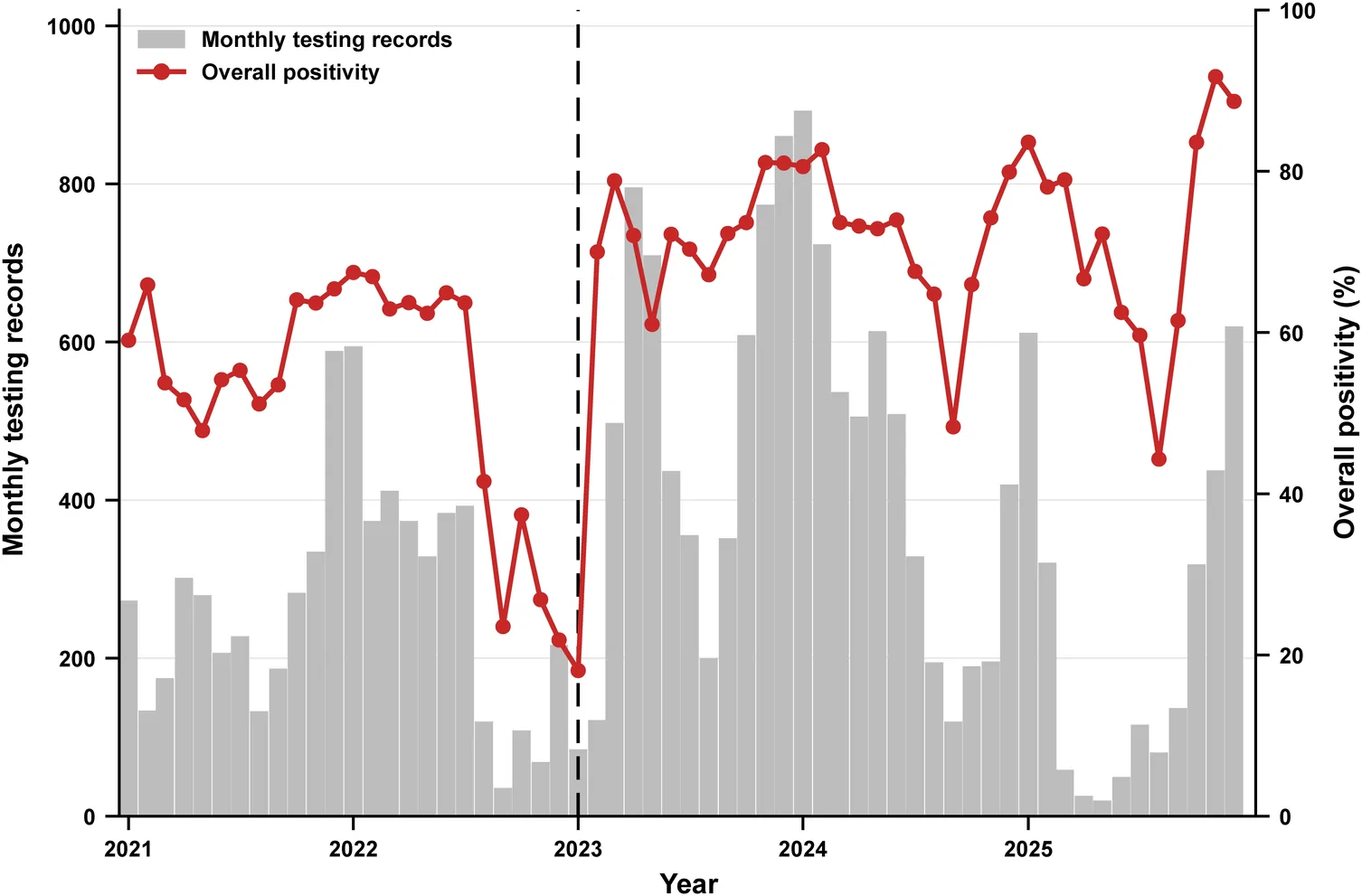
Monthly testing volume and overall respiratory pathogen positivity from January 2021 to December 2025. The grey bars show the monthly number of pediatric respiratory pathogen testing records, and the red line shows the overall positivity rate for at least one of the 13 main respiratory pathogen categories. The vertical dashed line indicates the start of the post-NPI period in January 2023. NPI, non-pharmaceutical intervention.

**Table 1:** Baseline characteristics and respiratory pathogen detection profiles of pediatric testing records during the NPI and post-NPI periods.

Characteristic	Overall (*N* = 20,250)	NPI period (*N* = 6,490)	Post-NPI period (*N* = 13,760)	*P* value
Age, years, median (IQR)	2.00 (0.75–4.00)	2.00 (0.67–4.00)	2.00 (0.75–4.00)	<0.001
Sex				0.013
Male	11,191 (55.3)	3,669 (56.5)	7,522 (54.7)	
Female	9,059 (44.7)	2,821 (43.5)	6,238 (45.3)	
Age group				<0.001
<1 year	5,961 (29.4)	2,058 (31.7)	3,903 (28.4)	
1–<3 years	5,115 (25.3)	1,751 (27.0)	3,364 (24.4)	
3–<6 years	6,112 (30.2)	1,877 (28.9)	4,235 (30.8)	
6–14 years	3,062 (15.1)	804 (12.4)	2,258 (16.4)	
Season				<0.001
Spring	5,608 (27.7)	1,860 (28.7)	3,748 (27.2)	
Summer	3,708 (18.3)	1,453 (22.4)	2,255 (16.4)	
Autumn	4,124 (20.4)	1,007 (15.5)	3,117 (22.7)	
Winter	6,810 (33.6)	2,170 (33.4)	4,640 (33.7)	
School-term status				<0.001
Vacation	3,379 (16.7)	1,389 (21.4)	1,990 (14.5)	
In-school term	16,871 (83.3)	5,101 (78.6)	11,770 (85.5)	
Overall detection indicators				
Overall positivity	14,191 (70.1)	3,813 (58.8)	10,378 (75.4)	<0.001
Co-detection	2,477 (12.2)	391 (6.0)	2,086 (15.2)	<0.001
Composite influenza A	1,433 (7.1)	122 (1.9)	1,311 (9.5)	<0.001
Pathogen-specific positivity				
InfA-NFC	187 (0.9)	8 (0.1)	179 (1.3)	<0.001
09H1	552 (2.7)	1 (0.0)	551 (4.0)	<0.001
H3	695 (3.4)	113 (1.7)	582 (4.2)	<0.001
HPIV	1,547 (7.6)	298 (4.6)	1,249 (9.1)	<0.001
HMPV	1,669 (8.2)	402 (6.2)	1,267 (9.2)	<0.001
Mp	1,884 (9.3)	386 (5.9)	1,498 (10.9)	<0.001
HRSV	3,084 (15.2)	812 (12.5)	2,272 (16.5)	<0.001
HADV	1,242 (6.1)	476 (7.3)	766 (5.6)	<0.001
HRV	4,859 (24.0)	1,404 (21.6)	3,455 (25.1)	<0.001
Boca	299 (1.5)	76 (1.2)	223 (1.6)	0.016
Ch	153 (0.8)	39 (0.6)	114 (0.8)	0.097
InfB	432 (2.1)	162 (2.5)	270 (2.0)	0.016
HCOV	284 (1.4)	43 (0.7)	241 (1.8)	<0.001

Data are shown as *n* (%) unless otherwise indicated. For grouped categorical variables, the *P* value is shown on the parent row. Composite influenza A was defined as positivity for any influenza A target at the record level, including InfA-NFC, 09H1, or H3, and was used only for descriptive summaries, not as an additional category in the main analyses. Component counts may not sum exactly to the composite count because a record could be positive for more than one influenza A component. Pathogen abbreviations and full pathogen names are provided in Supplementary Table S1. IQR, interquartile range; NPI, non-pharmaceutical intervention. *P* values compare the NPI and post-NPI periods. Continuous age is presented as median (interquartile range) and was compared using the Mann–Whitney *U* test.

Mean annual testing volume was 3,245.0 records during the NPI period and 4,586.7 during the post-NPI period, representing a 41.3% increase. Over the same periods, mean annual pediatric and neonatal inpatient admissions increased from 7,362.0 to 8,275.7, representing a 12.4% increase. The number of testing records per 100 inpatient admissions increased from 44.08 to 55.42, a relative increase of 25.7% (Supplementary Table S2).

### 3.2 Overall positivity and pathogen detection profile

Overall positivity for at least one of the 13 main respiratory pathogen categories was 70.1% (14,191/20,250). The overall positivity rate increased from 58.8% (3,813/6,490) during the NPI period to 75.4% (10,378/13,760) during the post-NPI period (*P* < 0.001). Co-detection of two or more pathogen categories was observed in 12.2% of records overall and increased from 6.0% (391/6,490) to 15.2% (2,086/13,760) after the lifting of NPIs (*P* < 0.001). Human rhinovirus was the most frequently detected pathogen category 24.0% (4,859/20,250), followed by human respiratory syncytial virus 15.2% (3,084/20,250), Mycoplasma pneumoniae 9.3% (1,884/20,250), human metapneumovirus 8.2% (1,669/20,250), human parainfluenza virus 7.6% (1,547/20,250), and human adenovirus 6.1% (1,242/20,250). The composite influenza A variable was positive in 7.1% of records, but it was used only for descriptive purposes and was not included as an additional category in the main 13-pathogen analyses. Overall, 29.9% (6,059/20,250) of testing records were negative for all 13 main pathogen categories, including 41.2% (2,677/6,490) during the NPI period and 24.6% (3,382/13,760) during the post-NPI period.

In the RSV-focused age-stratified supplementary analysis, children younger than 2 years accounted for 1,854 of 3,084 RSV-positive records (60.1%). Overall RSV positivity was higher among children aged <2 years than among those aged ≥2 years (21.0% vs. 10.8%; RR, 1.94; 95% CI, 1.82–2.08; *P* < 0.001). This excess was observed in both the NPI period (18.7% vs. 7.1%; RR, 2.63; 95% CI, 2.28–3.03; *P* < 0.001) and the post-NPI period (22.2% vs. 12.4%; RR, 1.79; 95% CI, 1.66–1.93; *P* < 0.001). In finer age categories, overall RSV positivity was 19.7% in children aged 0–<6 months, 23.1% in those aged 6–<12 months, 21.1% in those aged 12–<24 months, and 10.8% in those aged ≥24 months. Period-specific comparisons are provided in Supplementary Table S3.

### 3.3 Changes in pathogen detection risks between periods

Compared with the NPI period, the post-NPI period showed increased detection risks for most pathogen categories after false discovery rate correction (Table 2). Increased detection risks were also observed for influenza A virus not further classified by this panel (RR, 10.55; 95% CI, 5.20–21.42), seasonal human coronaviruses (RR, 2.64; 95% CI, 1.91–3.65), seasonal influenza A(H3N2) virus (RR, 2.43; 95% CI, 1.99–2.97), human parainfluenza virus (RR, 1.98; 95% CI, 1.75–2.24), Mycoplasma pneumoniae (RR, 1.83; 95% CI, 1.64–2.04), and human metapneumovirus (RR, 1.49; 95% CI, 1.33–1.66). Human respiratory syncytial virus, human rhinovirus, and human bocavirus also showed significant increases after the lifting of NPIs, with RRs of 1.32 (95% CI, 1.22–1.42), 1.16 (95% CI, 1.10–1.23), and 1.38 (95% CI, 1.07–1.79), respectively. In contrast, human adenovirus decreased from 7.3% to 5.6% (RR, 0.76; 95% CI, 0.68–0.85), and influenza B virus decreased from 2.5% to 2.0% (RR, 0.79; 95% CI, 0.65–0.95). Chlamydia spp. showed no statistically significant change after FDR correction.

**Table 2:** Changes in detection risks of 13 main respiratory pathogen categories between the NPI and post-NPI periods.

Pathogen	NPI period (*N* = 6,490)	Post-NPI period (*N* = 13,760)	RR (95% CI)	*P* value	FDR-adjusted *P* value
InfA-NFC	8 (0.1)	179 (1.3)	10.55 (5.20–21.42)	<0.001	<0.001
09H1	1 (0.0)	551 (4.0)	259.88 (36.55–1,847.87)	<0.001	<0.001
H3	113 (1.7)	582 (4.2)	2.43 (1.99–2.97)	<0.001	<0.001
HPIV	298 (4.6)	1,249 (9.1)	1.98 (1.75–2.24)	<0.001	<0.001
HMPV	402 (6.2)	1,267 (9.2)	1.49 (1.33–1.66)	<0.001	<0.001
Mp	386 (5.9)	1,498 (10.9)	1.83 (1.64–2.04)	<0.001	<0.001
HRSV	812 (12.5)	2,272 (16.5)	1.32 (1.22–1.42)	<0.001	<0.001
HADV	476 (7.3)	766 (5.6)	0.76 (0.68–0.85)	<0.001	<0.001
HRV	1,404 (21.6)	3,455 (25.1)	1.16 (1.10–1.23)	<0.001	<0.001
Boca	76 (1.2)	223 (1.6)	1.38 (1.07–1.79)	0.016	0.018
Ch	39 (0.6)	114 (0.8)	1.38 (0.96–1.98)	0.097	0.097
InfB	162 (2.5)	270 (2.0)	0.79 (0.65–0.95)	0.016	0.018
HCOV	43 (0.7)	241 (1.8)	2.64 (1.91–3.65)	<0.001	<0.001

Values in the NPI and post-NPI columns are positive records, *n* (%). Risk ratios were calculated as the positivity rate in the post-NPI period divided by the positivity rate in the NPI period. FDR-adjusted *P* values were calculated using the Benjamini–Hochberg method across the 13 main pathogen categories. The very large RR and wide confidence interval for 09H1 reflect sparse detections during the NPI period (1/6,490 records) compared with the post-NPI period (551/13,760 records), indicating a marked post-NPI increase with limited precision of the RR estimate. Composite influenza A, defined as positivity for InfA-NFC, 09H1, or H3, was excluded from this analysis to avoid double counting with influenza A-related categories. CI, confidence interval; FDR, false discovery rate; NPI, non-pharmaceutical intervention; RR, risk ratio.

### 3.4 Interrupted time-series analysis

In a single-trend model without interruption terms, overall positivity increased by an average of 0.49 percentage points per month over the 5-year study period (95% CI, 0.38–0.60; *P* < 0.001). In the segmented model with January 2023 as the interruption point, neither the immediate level change (+14.77 percentage points; *P* = 0.065) nor the slope change (+0.53 percentage points per month; *P* = 0.407) reached statistical significance, and the joint test of the level and slope changes was also not significant (*P* = 0.079). A grouped-binomial sensitivity model yielded a consistent result (joint *P* = 0.158; Supplementary Table S4). These findings indicate that overall positivity followed a long-term increasing trend over the 5-year study period, but no statistically significant independent level or slope change was observed at the January 2023 interruption point.

Against this overall temporal background, pathogen-specific interrupted time-series analysis showed heterogeneous temporal patterns across pathogens (Table 3 and Figure 3). In the primary model, positive immediate level-change estimates in monthly positivity were observed for human metapneumovirus (level change, 8.83 percentage points; 95% CI, 2.21–15.44; FDR-adjusted *P* = 0.058), influenza A H1N1 (2009) virus (5.10 percentage points; 95% CI, 0.88–9.31; FDR-adjusted *P* = 0.077), human respiratory syncytial virus (11.72 percentage points; 95% CI, 1.42–22.03; FDR-adjusted *P* = 0.084), human parainfluenza virus (8.46 percentage points; 95% CI, 0.36–16.56; FDR-adjusted *P* = 0.101), and seasonal human coronaviruses (1.92 percentage points; 95% CI, 0.03–3.82; FDR-adjusted *P* = 0.101). However, none of these positive immediate level-change estimates remained statistically significant after FDR correction.

**Table 3:** Interrupted time-series analysis of monthly positivity rates for 13 main respiratory pathogen categories after the lifting of COVID-19-related NPIs.

Pathogen	Positive records, *n*	Level change, pp (95% CI)	Level FDR adjusted *P* value	Trend change, pp/month (95% CI)	Trend FDR-adjusted *P* value
InfA-NFC	187	0.38 (−0.85 to 1.61)	0.643	0.01 (−0.06 to 0.08)	1.000
09H1	552	5.10 (0.88 to 9.31)	0.077	−0.12 (−0.35 to 0.12)	0.744
H3	695	−4.70 (−15.75 to 6.35)	0.584	0.02 (−0.79 to 0.83)	1.000
HPIV	1,547	8.46 (0.36 to 16.56)	0.101	0.01 (−0.46 to 0.49)	1.000
HMPV	1,669	8.83 (2.21 to 15.44)	0.058	0.17 (−0.36 to 0.71)	0.849
Mp	1,884	4.84 (−4.38 to 14.05)	0.537	−0.55 (−1.00 to −0.09)	0.117
HRSV	3,084	11.72 (1.42 to 22.03)	0.084	0.51 (−0.28 to 1.30)	0.544
HADV	1,242	−13.00 (−20.13 to −5.87)	0.005	−0.27 (−0.70 to 0.15)	0.544
HRV	4,859	−2.04 (−11.43 to 7.34)	0.725	1.07 (0.63 to 1.50)	<0.001
Boca	299	0.86 (−1.59 to 3.32)	0.636	0.02 (−0.08 to 0.12)	1.000
Ch	153	0.03 (−0.67 to 0.74)	0.929	0.04 (−0.01 to 0.08)	0.524
InfB	432	1.65 (−1.68 to 4.99)	0.537	−0.10 (−0.37 to 0.18)	0.849
HCOV	284	1.92 (0.03 to 3.82)	0.101	0.00 (−0.10 to 0.10)	1.000

Segmented regression models used monthly positivity rates as outcomes. January 2023 was treated as the start of the post-NPI period. Models included time, intervention status, time after intervention, and month fixed effects, and were weighted by the monthly number of testing records. Newey–West robust standard errors with lag 1 were used in the primary model. Level change represents the immediate change in monthly positivity after the lifting of NPIs, and trend change represents the change in monthly slope after the intervention. For compact main-text presentation, FDR-adjusted *P* values are shown; additional robustness and sensitivity analyses are provided in Supplementary Tables S7A–C. CI, confidence interval; FDR, false discovery rate; ITS, interrupted time series; NPI, non-pharmaceutical intervention; pp, percentage points.

**Figure 3:**
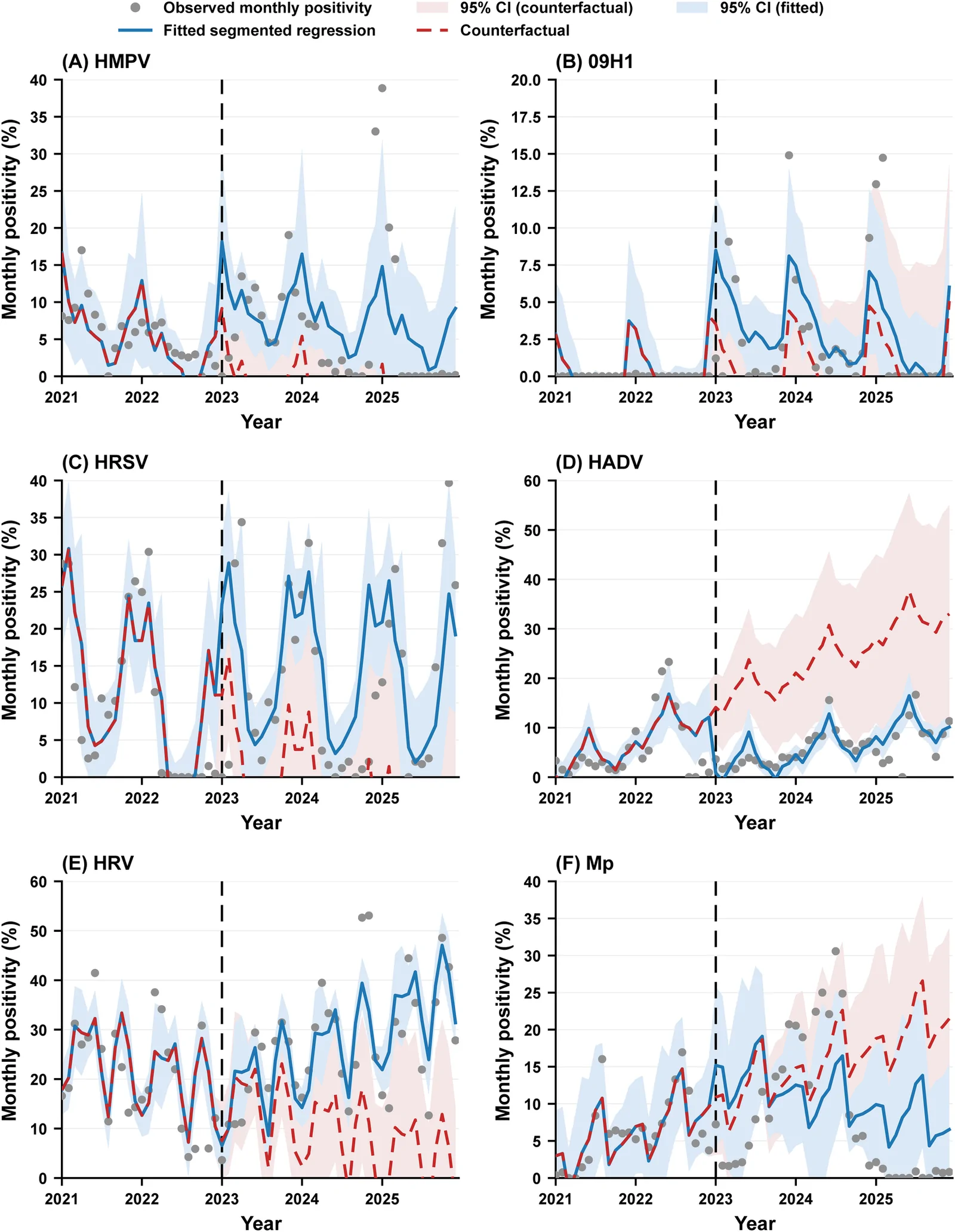
Interrupted time-series trends of selected pediatric respiratory pathogens before and after the lifting of COVID-19-related NPIs. Monthly positivity rates are shown for six selected respiratory pathogen categories: **(A)** HMPV, **(B)** 09H1, **(C)** HRSV, **(D)** HADV, **(E)** HRV, and **(F)** Mp. Grey points indicate observed monthly positivity rates. Blue solid lines and blue shaded areas indicate fitted segmented regression trends and 95% confidence intervals. Red dashed lines and red shaded areas indicate counterfactual projections based on the pre-intervention trend and their 95% confidence intervals. The vertical dashed line indicates the start of the post-NPI period in January 2023. *Y*-axis scales vary across panels to improve visualization of pathogen-specific temporal patterns. 09H1, influenza A H1N1 (2009) virus; HADV, human adenovirus; HMPV, human metapneumovirus; HRV, human rhinovirus; HRSV, human respiratory syncytial virus; ITS, interrupted time series; Mp, *Mycoplasma pneumoniae*; NPI, non-pharmaceutical intervention.

In contrast, human adenovirus showed an immediate level decrease that remained statistically significant after FDR correction (level change, −13.00 percentage points; 95% CI, −20.13 to −5.87; FDR-adjusted *P* = 0.005). Human rhinovirus showed a significant positive trend change after the January 2023 interruption, with the monthly slope increasing by 1.07 percentage points per month relative to the NPI-period slope (95% CI, 0.63–1.50; FDR-adjusted *P* < 0.001). The corresponding post-NPI slope was +0.64 percentage points per month (95% CI, 0.37–0.91; *P* < 0.001). Mycoplasma pneumoniae showed a negative trend-change estimate in the primary model (−0.55 percentage points per month; 95% CI, −1.00 to −0.09), but this association did not remain statistically significant after FDR correction (FDR-adjusted *P* = 0.117).

### 3.5 Associations with meteorological factors, AQI, and school-term status

Multivariable logistic regression models were fitted to evaluate factors associated with pathogen positivity after adjustment for age group, sex, and pandemic phase (Figure 4 and Supplementary Table S5A). Across the 60-month study period, the monthly mean AQI had a mean of 56.02, median of 51.91, IQR of 40.70–63.52, and range of 26.50–106.81, providing contextual distribution information for the AQI variable included in these models. Higher monthly mean temperature was associated with lower positivity of human respiratory syncytial virus (aOR, 0.86 per 1 °C increase; 95% CI, 0.81–0.91), influenza A H1N1 (2009) virus (aOR, 0.84; 95% CI, 0.76–0.94), and influenza B virus (aOR, 0.83; 95% CI, 0.74–0.93). In contrast, higher temperature was associated with higher positivity of human parainfluenza virus (aOR, 1.09; 95% CI, 1.02–1.16) and human bocavirus (aOR, 1.13; 95% CI, 1.04–1.22). These associations remained significant after FDR correction.

**Figure 4:**
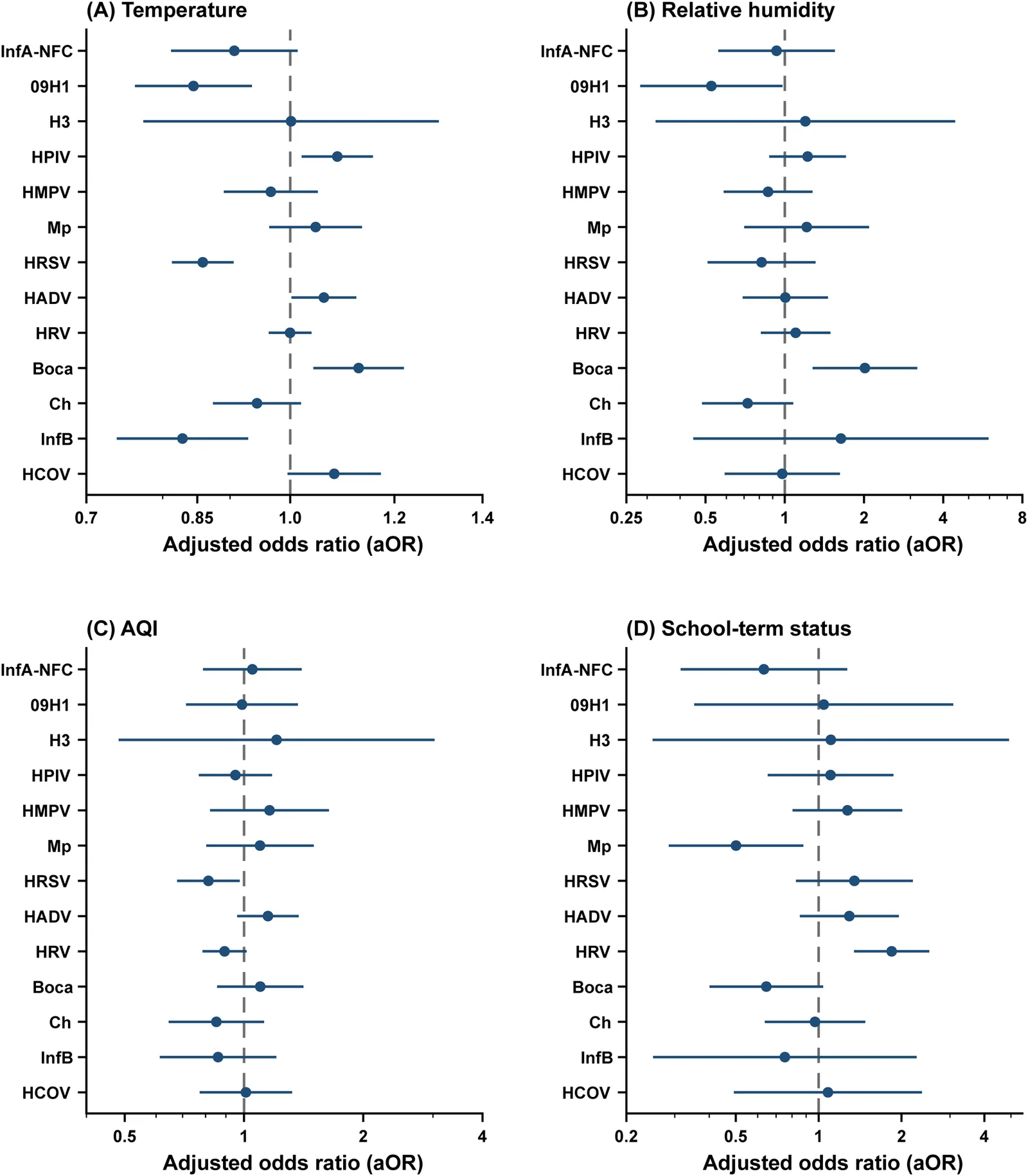
Multivariable associations of meteorological factors, air quality, and school-term status with positivity of 13 main respiratory pathogen categories. Separate multivariable logistic regression models were fitted for each of the 13 main respiratory pathogen categories. The figure shows adjusted odds ratios and 95% confidence intervals for **(A)** monthly mean temperature, **(B)** monthly mean relative humidity, **(C)** monthly mean air quality index, and **(D)** school-term status. Temperature was modeled per 1 °C increase, relative humidity per 10% increase, AQI per 10-unit increase, and school-term status as in-school term vs. vacation. Models were adjusted for age group, sex, and pandemic phase, with cluster-robust standard errors estimated at the year-month level. The vertical dashed line indicates an adjusted odds ratio of 1.0. *X*-axis scales vary across panels to improve visualization of factor-specific effect estimates. 09H1, influenza A H1N1 (2009) virus; AQI, air quality index; aOR, adjusted odds ratio; Boca, human bocavirus; Ch, *Chlamydia* spp.; CI, confidence interval; HADV, human adenovirus; H3, seasonal influenza A (H3N2) virus; HCOV, seasonal human coronaviruses; HMPV, human metapneumovirus; HPIV, human parainfluenza virus; HRV, human rhinovirus; HRSV, human respiratory syncytial virus; InfA-NFC, influenza A virus not further classified by this panel, defined as positivity for the generic influenza A target without classification as 09H1 or H3; InfB, influenza B virus; Mp, *Mycoplasma pneumoniae*.

Higher monthly mean relative humidity was mainly associated with increased human bocavirus positivity (aOR, 2.02 per 10% increase; 95% CI, 1.28–3.19; FDR-adjusted *P* = 0.035). No robust association was observed between AQI and any pathogen category after FDR correction. School-term status was associated with increased human rhinovirus positivity compared with vacation periods (aOR, 1.84; 95% CI, 1.34–2.52; FDR-adjusted *P* = 0.002). Mycoplasma pneumoniae showed lower odds of positivity during school terms in the primary model (aOR, 0.50; 95% CI, 0.29–0.88; *P* = 0.016), but this association did not remain statistically robust after FDR correction.

### 3.6 Phase-interaction analysis

To assess whether associations between relevant factors and pathogen positivity differed between the NPI and post-NPI study periods, we further conducted prespecified phase interaction analyses. Of 52 prespecified interaction tests, 51 were estimable; because positive 09H1 events were sparse during the NPI period, the school term × phase interaction could not be reliably estimated (Supplementary Table S6).

For HPIV, the temperature × phase interaction was statistically significant (interaction OR, 1.22; 95% CI, 1.12–1.33; *q* < 0.001), with phase-specific associations of aOR 0.96 (95% CI, 0.93–0.99) during the NPI period and aOR 1.17 (95% CI, 1.08–1.27) during the post-NPI period. For H3, the school term × phase interaction was statistically significant (interaction OR, 21.48; 95% CI, 3.57–129.32; *q* = 0.021), with phase-specific aORs of 0.58 (95% CI, 0.25–1.37) and 12.53 (95% CI, 2.61–60.23), respectively. For Ch, the relative humidity × phase interaction was statistically significant (interaction OR, 0.34; 95% CI, 0.17–0.67; *q* = 0.033), with phase-specific aORs of 1.62 (95% CI, 0.95–2.75) and 0.55 (95% CI, 0.37–0.83), respectively.

Overall, only the temperature × phase interaction for HPIV, the school term × phase interaction for H3, and the relative humidity × phase interaction for Ch remained statistically significant after FDR correction; none of the other estimable interactions reached statistical significance.

### 3.7 Age-stratified school-term analysis

Age-stratified logistic regression analyses suggested pathogen-specific and age-dependent associations between school-term status and pathogen positivity (Figure 5 and Supplementary Table S5B). After FDR correction within each age stratum, human rhinovirus remained significantly associated with higher positivity during school terms across all three age strata: <3 years (aOR, 1.69; 95% CI, 1.24–2.31; FDR-adjusted *P* = 0.012), 3–<6 years (aOR, 2.15; 95% CI, 1.53–3.03; FDR-adjusted *P* < 0.001), and 6–14 years (aOR, 2.61; 95% CI, 1.76–3.85; FDR-adjusted *P* < 0.001).

**Figure 5:**
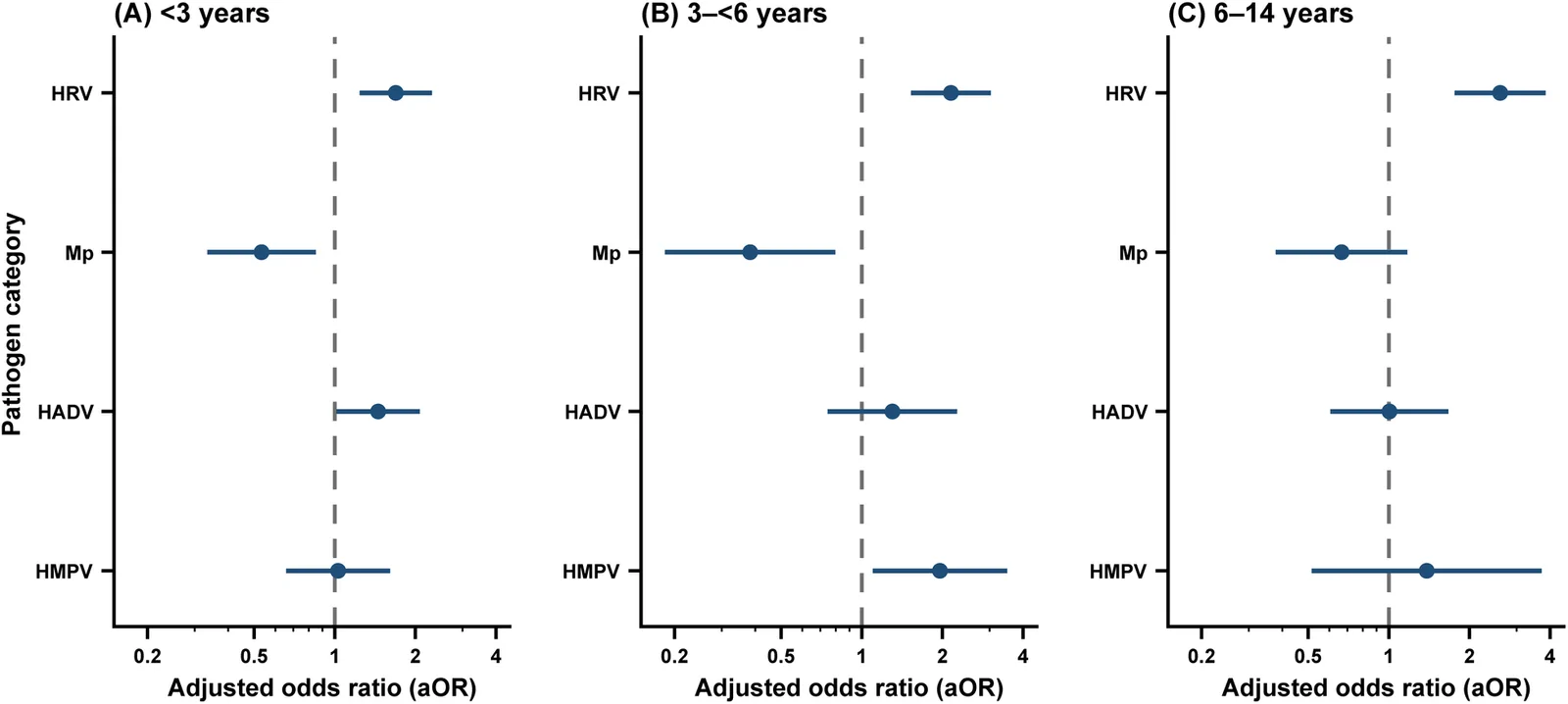
Age-stratified associations between school-term status and selected respiratory pathogen positivity. Age-stratified multivariable logistic regression models were fitted to evaluate the association between school-term status and selected respiratory pathogen positivity in three age strata: **(A)** <3 years, **(B)** 3–<6 years, and **(C)** 6–14 years. School-term status was modeled as in-school term vs. vacation. Within each age stratum, models were adjusted for sex, pandemic phase, and monthly mean meteorological and air-quality variables, including temperature, relative humidity, and AQI, with cluster-robust standard errors estimated at the year-month level. Points indicate adjusted odds ratios, and horizontal lines indicate 95% confidence intervals. The vertical dashed line indicates an adjusted odds ratio of 1.0. AQI, air quality index; aOR, adjusted odds ratio; CI, confidence interval; HADV, human adenovirus; HMPV, human metapneumovirus; HRV, human rhinovirus; Mp, *Mycoplasma pneumoniae*.

For selected other pathogens shown in Figure 5, nominal associations were observed for Mycoplasma pneumoniae in children aged <3 years (aOR, 0.53; 95% CI, 0.33–0.85; *P* = 0.008) and 3–<6 years (aOR, 0.38; 95% CI, 0.18–0.80; *P* = 0.010), human adenovirus in children aged <3 years (aOR, 1.45; 95% CI, 1.01–2.08; *P* = 0.041), and human metapneumovirus in children aged 3–<6 years (aOR, 1.96; 95% CI, 1.10–3.49; *P* = 0.023); none of these associations remained statistically significant after FDR correction.

### 3.8 Supplementary and sensitivity analyses

To assess the robustness of the main findings to time-series model specification, covariate adjustment, and repeated testing of the same child, we performed multiple sensitivity analyses and additional co-detection analyses. ITS robustness analyses yielded broadly consistent directions for the main pathogen-specific signals, including the positive trend change and positive post-NPI slope of human rhinovirus and the immediate decrease of human adenovirus (Supplementary Tables S7A–C). The core predictors in the multivariable logistic regression models showed no evidence of severe collinearity, with the highest variance inflation factor being 4.28 (Supplementary Table S5C), and season-adjusted sensitivity models showed broadly similar directions for the main Figure 4 findings (Supplementary Table S5D). Supplementary co-detection analyses showed that the risk of co-detection was higher in the post-NPI period than in the NPI period (RR, 2.52; 95% CI, 2.27–2.79; Supplementary Table S8A). Weighted ITS analysis showed an immediate increase of 7.80 percentage points in the monthly co-detection rate at the January 2023 interruption point (95% CI, 2.92–12.68; *P* = 0.002; Supplementary Table S8B). A binomial ITS sensitivity model yielded a consistent immediate increase in co-detection (OR, 2.76; 95% CI, 2.13–3.57; *P* < 0.001; Supplementary Table S8C).

In the first-record-only sensitivity analysis, 16,663 unique children were included, including 5,886 in the NPI period and 10,777 in the post-NPI period. Overall positivity remained higher in the post-NPI period than in the NPI period: 74.1% (7,984/10,777) vs. 58.4% (3,436/5,886) (RR, 1.27; 95% CI, 1.24–1.30; *P* < 0.001), as did co-detection: 14.4% (1,547/10,777) vs. 6.0% (353/5,886) (RR, 2.39; 95% CI, 2.14–2.67; *P* < 0.001). The direction of the period comparison was consistent with the main analysis across all 13 pathogen categories, with broadly similar effect sizes. The FDR-adjusted statistical significance of Boca and Ch changed after restricting the analysis to the first record for each child, but the direction of effect remained unchanged for both (Supplementary Table S9). Overall, none of the sensitivity analyses altered the direction of the main findings, and the results were broadly consistent with the main analysis.

## 4 Discussion

### 4.1 Principal findings

This 5-year hospital-based laboratory surveillance study identified marked changes in pediatric respiratory pathogen detection across the NPI and post-NPI periods, with distinct temporal patterns across pathogens. Overall positivity was higher in the post-NPI period than in the NPI period (75.4% vs. 58.8%), and co-detection was also more frequent (15.2% vs. 6.0%). At the aggregate level, overall positivity showed a significant long-term increasing trend over the 5-year study period, whereas segmented analysis using January 2023 as the interruption point showed no statistically significant immediate level or slope change. At the pathogen-specific level, ITS analyses revealed distinct temporal trajectories across pathogens, with human adenovirus showing a robust immediate decrease and human rhinovirus showing a significant positive trend change and a positive post-NPI slope. Associations with environmental factors and school-calendar status were likewise pathogen-specific, and phase-interaction analyses further showed that selected associations with temperature, relative humidity, and school-term status differed between the NPI and post-NPI periods. Overall, these findings demonstrate a complex temporal profile of pediatric respiratory pathogen detection characterized by a long-term aggregate trend, pathogen-specific changes, and associations with environmental and school-calendar factors.

### 4.2 Overall positivity, long-term trend, and co-detection across study periods

COVID-19-related NPIs substantially altered the circulation of common respiratory pathogens (7–11), and subsequent studies reported renewed circulation and altered epidemiological patterns of multiple respiratory pathogens following relaxation of these measures (12–16). In our study, overall positivity was higher in the post-NPI period than in the NPI period. Further analysis of the overall temporal trend showed that overall positivity increased by an average of 0.49 percentage points per month over the 5-year study period, whereas segmented analysis with January 2023 as the interruption point did not show significant changes in the overall level or trend. These findings place the higher overall positivity observed in the post-NPI period within a broader long-term upward trend and suggest that aggregate measures may not fully capture heterogeneous temporal dynamics across individual pathogens.

Co-detection also increased markedly in the post-NPI period, and this increase was supported by both the period comparison and weighted ITS analysis. This pattern may reflect greater temporal overlap in the circulation of multiple respiratory pathogens, thereby increasing the likelihood of detecting more than one pathogen in the same specimen. For hospital-based laboratory surveillance, co-detection patterns provide information beyond single-pathogen positivity and add complexity to etiological attribution and laboratory interpretation. Accordingly, considering both single-pathogen temporal trends and multi-pathogen co-detection patterns may provide a more complete characterization of pediatric respiratory pathogen activity in the post-NPI period.

### 4.3 Pathogen-specific reshaping of detection patterns

A central finding of this study is that pathogen detection patterns changed heterogeneously after the lifting of NPIs. Although many pathogen categories showed higher detection risks in the post-NPI period, the magnitude, timing, and stability of changes varied across pathogens. This pattern is consistent with reports of asynchronous respiratory virus resurgence and pathogen-specific recovery after relaxation of COVID-19-related restrictions (12, 14, 30). It supports the concept that the post-NPI period may represent pathogen-specific reshaping rather than a simple synchronized return to pre-pandemic seasonality. This distinction is important because aggregate respiratory pathogen positivity may obscure divergent trajectories among individual pathogens.

Human rhinovirus showed the most statistically robust trend-related finding in our ITS analysis, with a significant positive trend change after the January 2023 interruption and a positive post-NPI slope. This pattern is consistent with previous pediatric studies showing that rhinovirus activity was less suppressed by NPIs and could remain prominent or increase while several other respiratory viruses showed marked reductions or altered circulation (31–33). The positive association between school-term status and human rhinovirus positivity across age strata is also consistent with evidence that school sessions, holidays, and closures can influence respiratory infection activity in children (20, 34, 35). Thus, human rhinovirus may function as a sensitive indicator of renewed pediatric contact networks in the post-NPI period. Nevertheless, school-term status in this study was defined by calendar timing and should be interpreted as a population-level contextual indicator rather than a direct measure of individual school attendance, classroom exposure, or transmission pathway.

Several pathogens, including human metapneumovirus, influenza A H1N1 (2009) virus, human respiratory syncytial virus, human parainfluenza virus, and seasonal human coronaviruses, showed immediate level increases in the primary ITS model. Similar post-restriction shifts in human metapneumovirus, influenza, and other respiratory viruses have been reported after periods of reduced circulation or relaxation of COVID-19 restrictions (13, 36, 37). However, these signals in our study were attenuated after FDR correction, indicating lower statistical robustness under multiple-comparison control. These findings are still informative because they suggest transient post-NPI increases for several pathogens, while also showing that not all apparent rebounds were statistically stable after adjustment for multiple testing. For influenza A H1N1 (2009) virus, the large period-comparison RR and wide confidence interval mainly reflected sparse detections during the NPI period, indicating a marked post-NPI increase with limited precision of the RR estimate.

The RSV-focused supplementary analysis further highlights the translational relevance of age-specific surveillance, consistent with previous evidence that RSV contributes substantially to acute lower respiratory infection burden in young children and that RSV activity was altered during and after COVID-19-related restriction periods (2, 10–12). Although the primary age strata were designed to reflect childcare and schooling stages in China, reclassification into RSV prevention-relevant age windows showed that children younger than 2 years accounted for most RSV-positive records and had nearly twice the overall RSV detection risk observed in older children. The highest positivity was observed in the 6–<12-month and 12–<24-month groups, supporting the importance of infant- and toddler-focused RSV prevention and surveillance strategies. The stronger relative post-NPI increase among children aged ≥2 years also suggests broader age redistribution of RSV detection after the lifting of NPIs. Because this study was based on laboratory testing records, these findings should be interpreted as age-specific RSV detection patterns in a laboratory surveillance dataset and may inform future studies that link virological detection with hospitalization and severity outcomes.

Human adenovirus showed a contrasting pattern, with both a lower post-NPI detection risk and a robust immediate level decrease in ITS analysis. This divergence from HRV further highlights the pathogen-specific nature of post-NPI changes. This pattern underscores the value of local pathogen-specific laboratory surveillance, because decreases in selected pathogens may be hidden when respiratory pathogen activity is assessed only through aggregate positivity. Mycoplasma pneumoniae showed increased detection risk in the post-NPI period in the period comparison, but its negative trend change in the ITS model did not remain significant after FDR correction. This finding should be interpreted alongside recent reports of *Mycoplasma pneumoniae* resurgence after the relaxation of COVID-19 control measures (14, 15) and emerging discussions of its post-pandemic pediatric epidemiology (38, 39). The pattern observed here may reflect temporally uneven activity, supporting the need for longer observation windows and pathogen-specific modeling rather than relying solely on period-level comparisons.

### 4.4 Meteorological, AQI, and school-term associations

The associations observed between meteorological factors and pathogen positivity highlight the importance of considering environmental context when interpreting laboratory surveillance data. Higher temperature was associated with lower positivity of human respiratory syncytial virus, influenza A H1N1 (2009) virus, and influenza B virus, but higher positivity of human parainfluenza virus and human bocavirus. These divergent associations are consistent with evidence that viral seasonality and meteorological factors are linked to respiratory pathogen activity (17, 18), and that specific meteorological parameters may influence acute respiratory infections and respiratory virus activity in pathogen-dependent ways (40–42). Higher relative humidity was mainly associated with increased human bocavirus positivity, whereas AQI showed no robust association with any pathogen category after FDR correction. Evidence linking air pollution with respiratory viral infection and child respiratory health provides relevant context for including AQI in surveillance models (19, 43), although broader reviews also emphasize that air-quality effects vary across populations, pollutants, and settings (44, 45). These findings support the view that environmental variables may provide useful contextual information for pathogen-specific surveillance, even when they do not operate as uniform predictors across all respiratory pathogens.

Meteorological and AQI variables were linked at the monthly ecological level and should therefore be interpreted as contextual indicators rather than individual-level exposure measures. Temperature, humidity, seasonality, and human behavior are interrelated, making it difficult to isolate a single environmental factor as a causal determinant of pathogen detection. Our collinearity and season-adjusted sensitivity analyses suggested that the main associations were not driven by severe model instability. Accordingly, these models are most useful for identifying surveillance-relevant contextual associations that can help explain temporal variation in laboratory detection patterns.

Phase-interaction analyses further indicated that associations of environmental and school-calendar factors with pathogen positivity were not uniform across the NPI and post-NPI periods, but instead showed selective, pathogen-specific variation. Only a small subset of the prespecified interactions remained statistically significant after correction for multiple testing, suggesting that such between-period differences may be concentrated in specific pathogens and particular environmental or contact contexts. The changes in the direction of the associations of temperature with HPIV and relative humidity with Ch further suggest that environmental associations may vary by pathogen and study period. Previous studies have likewise shown that different respiratory pathogens exhibit distinct associations with meteorological factors such as temperature and humidity. For example, lower temperatures have often been associated with increased activity of RSV and influenza viruses, whereas associations with humidity vary across other respiratory viruses (17, 18, 40–42). The association between school-term status and H3 was more pronounced in the post-NPI period, while previous studies of school sessions, school holidays, school closures, and school reopening have shown that changes in child contact patterns associated with school terms and holidays are closely related to respiratory infection activity, particularly influenza transmission (20, 34, 35, 46–48). Together, these findings suggest that associations of environmental and school-calendar factors with respiratory pathogen detection may differ across study periods and should therefore be interpreted in the context of both the specific pathogen and the study period.

School-term status was associated with increased human rhinovirus positivity in the overall model and across all age strata. This result is plausible because school sessions and school holidays have been associated with seasonal respiratory infection activity and influenza transmission in children (20, 34, 35). Evidence from studies of school closures, school opening dates, and scheduled school breaks further supports the role of school calendars in shaping respiratory virus circulation (46–48). Studies evaluating planned school breaks or relaxation of school and daycare restrictions also show that changes in school-related contact structures can coincide with shifts in pediatric respiratory virus activity (36, 37, 49). Rather than serving as direct proof of school-based transmission, this finding is best interpreted as a school-calendar signal that may help identify periods of intensified pediatric respiratory pathogen circulation. The age-stratified analyses further suggested that school-term associations differed by pathogen and age group, supporting the need for age-sensitive interpretation of pediatric respiratory pathogen surveillance data. In practice, combining school-calendar information with laboratory surveillance may improve the timing and interpretation of pediatric respiratory pathogen monitoring.

The associations between school-term status and pathogen positivity should be interpreted as population-level school-calendar signals. School opening periods may increase contact intensity among kindergarten- and school-aged children and thereby influence broader community transmission networks. These networks may also affect infants and toddlers indirectly through household contacts, siblings, childcare environments, and community-level exposure. This interpretation supports the age-stratified findings while maintaining the ecological nature of the school-term variable.

### 4.5 Hospital-based laboratory surveillance implications and limitations

The main practical implication of this study is to support continuous hospital-based multi-pathogen laboratory surveillance by integrating pathogen-specific temporal trends, age stratification, the concurrent circulation of multiple pathogens, and relevant environmental and school-calendar context, thereby providing a more complete context for interpreting laboratory detection patterns. For example, an RSV-positive result may be interpreted in the context of contemporaneous age-specific and seasonal RSV activity, an HRV-positive result alongside current HRV activity and school-calendar patterns, and a co-detection in light of the concurrent circulation of the detected pathogens. Integrating these elements into routine laboratory surveillance may help identify pathogens undergoing change and pediatric populations requiring particular attention, while informing surveillance priorities, service preparedness, and interpretation of laboratory results in pediatric and neonatal care.

Although the present study focused on laboratory pathogen surveillance, emerging non-invasive approaches to respiratory functional assessment, including advanced ventilation MRI techniques (50, 51) and respiratory-motion monitoring technologies (52), may provide complementary information for the evaluation of respiratory function. Their potential roles in the broader assessment of pediatric respiratory disease warrant further investigation.

This study has three main limitations. First, it was a single-center hospital-based laboratory surveillance study involving pediatric and neonatal inpatients who underwent routine respiratory pathogen testing; therefore, temporal changes in patient composition and testing utilization may have influenced the observed detection patterns. Second, the routine 13-pathogen panel did not include SARS-CoV-2 and could not capture all respiratory pathogens; in addition, the dataset did not include individual-level information on prior SARS-CoV-2 infection, COVID-19 vaccination, post-COVID condition (long COVID), or detailed clinical characteristics and outcomes. These factors may be associated with temporal variation in pathogen detection and should be considered when interpreting panel-negative and co-detection findings. Third, meteorological variables, AQI, and school-term status were population-level contextual measures, while the January 2023 temporal boundary may also have been influenced by long-term trends, natural pathogen cycles, changes in testing utilization, and other concurrent factors. Future multicenter studies integrating broader pathogen testing, longitudinal laboratory surveillance, individual-level COVID-19-related information, and clinical outcomes are needed to further clarify these surveillance patterns and their clinical and public health relevance.

## 5 Conclusion

Over 5 years of continuous hospital-based laboratory surveillance, pediatric respiratory pathogen detection showed distinct overall and pathogen-specific patterns across the NPI and post-NPI periods, with higher overall positivity and co-detection rates in the post-NPI period. Pathogen-specific interrupted time-series analyses further revealed different temporal trajectories, including an immediate decrease in HADV and a significant positive trend change in HRV with a positive post-NPI slope. Age-stratified analyses and assessments of meteorological and school-calendar factors further showed that associations between pathogen detection, age, and contextual factors were pathogen-specific; phase-interaction analyses additionally indicated that the associations of temperature with HPIV, school-term status with H3, and relative humidity with Ch differed between the NPI and post-NPI periods. Taken together, these findings support continued hospital-based multi-pathogen laboratory surveillance integrating pathogen-specific temporal trends, age stratification, co-detection patterns, and environmental and school-calendar context to facilitate dynamic identification of pediatric respiratory pathogen activity, prioritization of surveillance targets, and pediatric service preparedness.

## Data Availability

The datasets analyzed in this study are not publicly available because they contain hospital-based laboratory testing records and are subject to institutional and ethical restrictions. Aggregated and de-identified data supporting the findings of this study are available from the corresponding author upon reasonable request and subject to institutional approval, ethical approval, and a data use agreement.
